# Supplementary material for: Enhancement of E. coli acyl-CoA synthetase FadD activity on medium chain fatty acids
Source: PeerJ. 2015 Jun 30;3:e1040. doi: 10.7717/peerj.1040 (PMC4493641; doi:10.7717/peerj.1040)

Trial 1

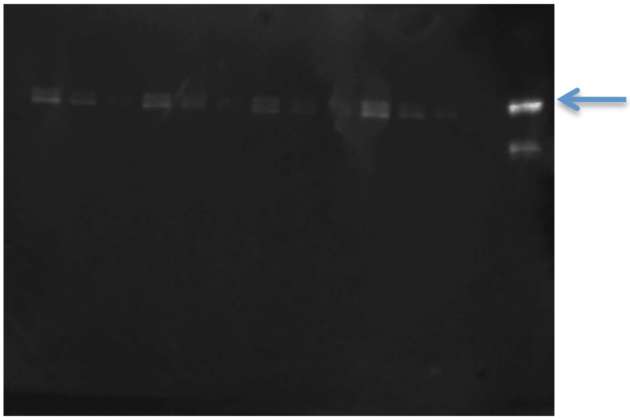

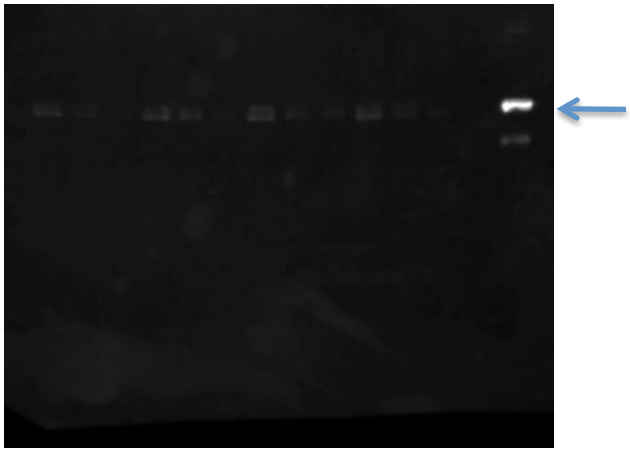

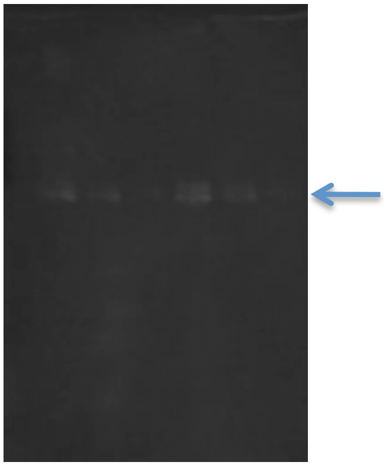

Trial 2

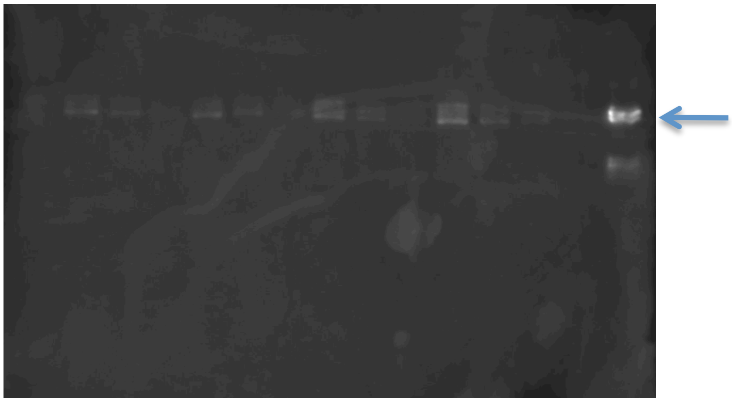

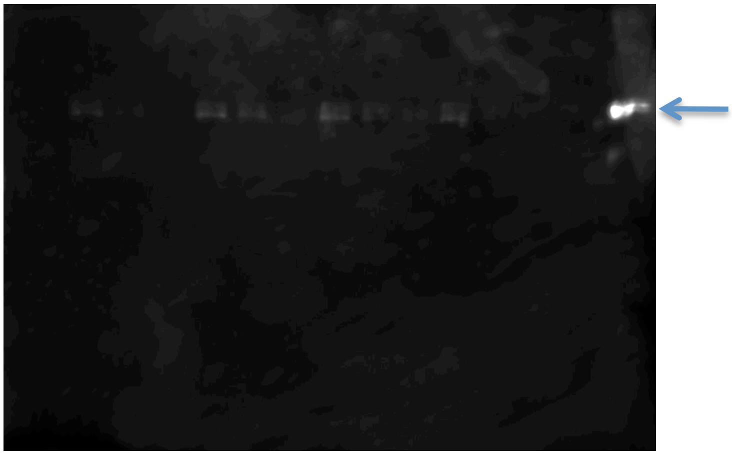

Trial 3

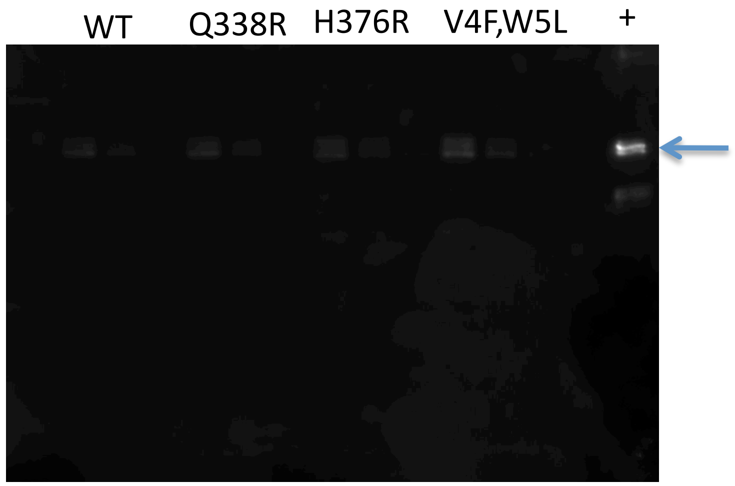

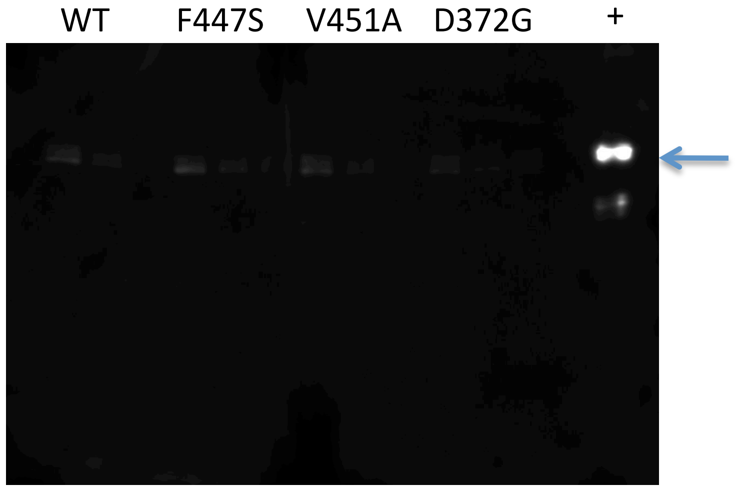

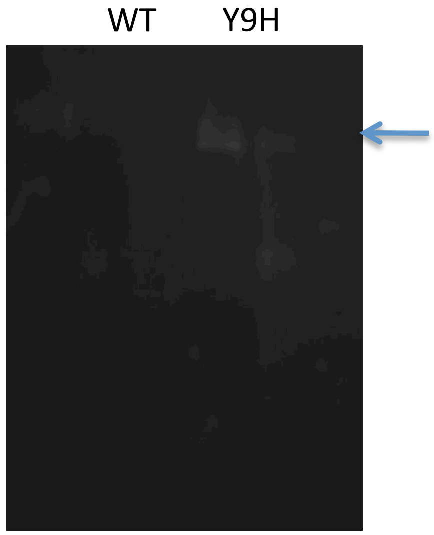

Trial 4

WT Q338R H376R V4F,W5L +

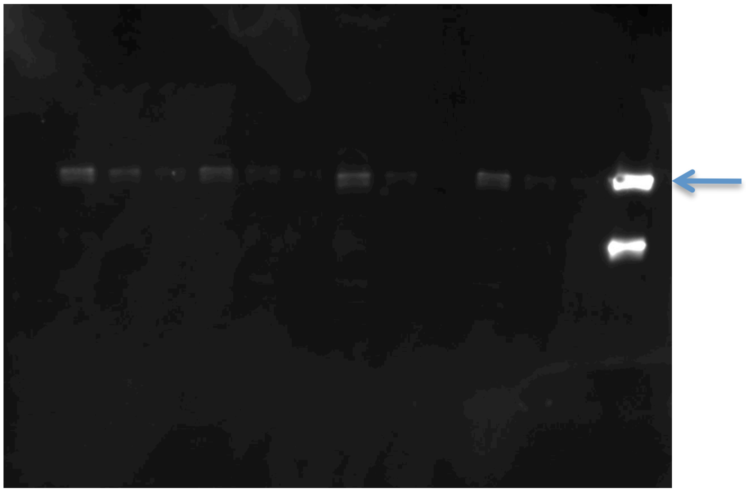

WT F447S V451A D372G +

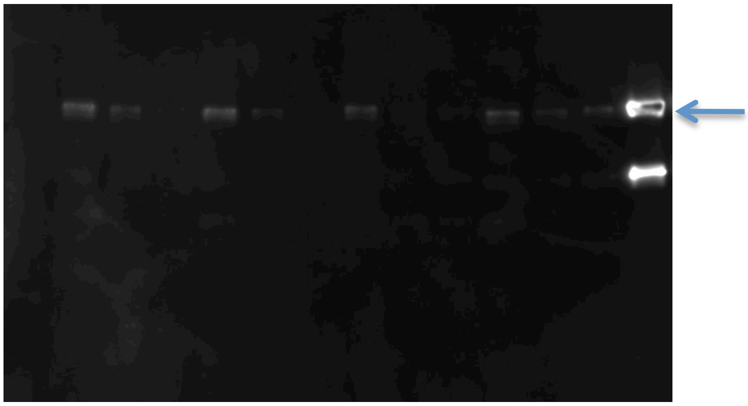

WT Y9H

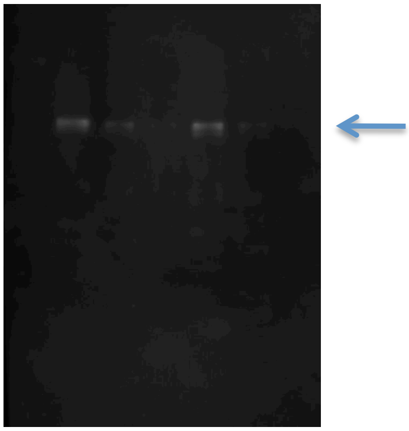

Trial 5

WT Q338R H376R V4F,W5L +

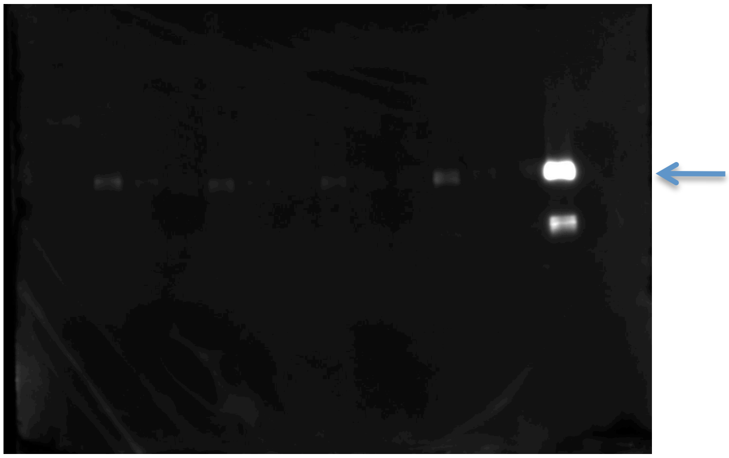

WT F447S V451A D372G +

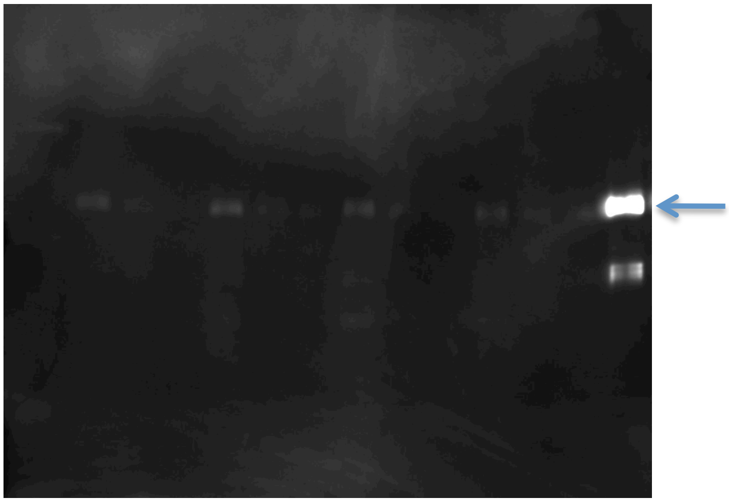

WT Y9H

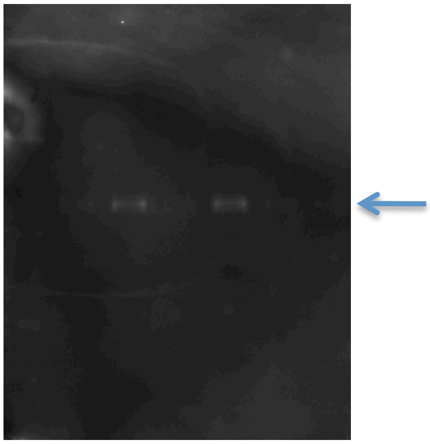

Supplement: Figure S1 — Western blots containing extracts from ΔfadR strains expressing the indicated His-tagged FadD mutants from plasmid pETDuet-1 and grown on octanoate minimal medium as described in materials and methods. Blots used an antibody to the 6x-His tag. Blots contain undiluted extracts, extracts diluted 1:2, and extracts diluted 1:4 for each strain (from left to right). (+) indicates positive control lane containing partially purified wt FadD. Arrows indicate full-length FadD in all cases. Results of quantification as indicated in materials and methods are shown in Fig. 1D. [file peerj-03-1040-s001.pdf]
