# Supplementary material for: Enhancement of E. coli acyl-CoA synthetase FadD activity on medium chain fatty acids
Source: PeerJ. 2015 Jun 30;3:e1040. doi: 10.7717/peerj.1040 (PMC4493641; doi:10.7717/peerj.1040)
Supplement: Supplemental Information 6 [file peerj-03-1040-s006.pdf]

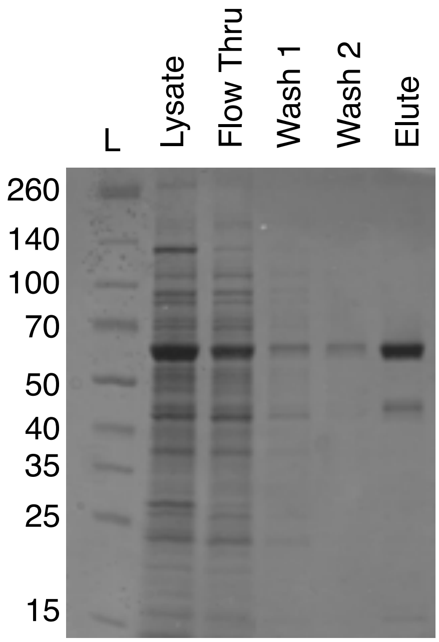

Figure S1: Sample partial purifications of C-terminally His_6-tagged FadD. Lanes contain: (L) Spectra Multicolor Broad Range Protein Ladder (Thermo Scientific) with the molecular weight (kDa) of each band in the ladder next to the gel, (Lystate) lysate fraction from Ni-NTA partial purification (Materials and Methods), (Flow Thru), flow thru fraction from the Ni-NTA partial purification (Materials and Methods), (Wash 1 and 2) wash fractions from the Ni-NTA partial purification (Materials and Methods), and (Elute) pooled elutant fractions from the Ni-NTA partial purification of wild-type FadD (Materials and Methods).

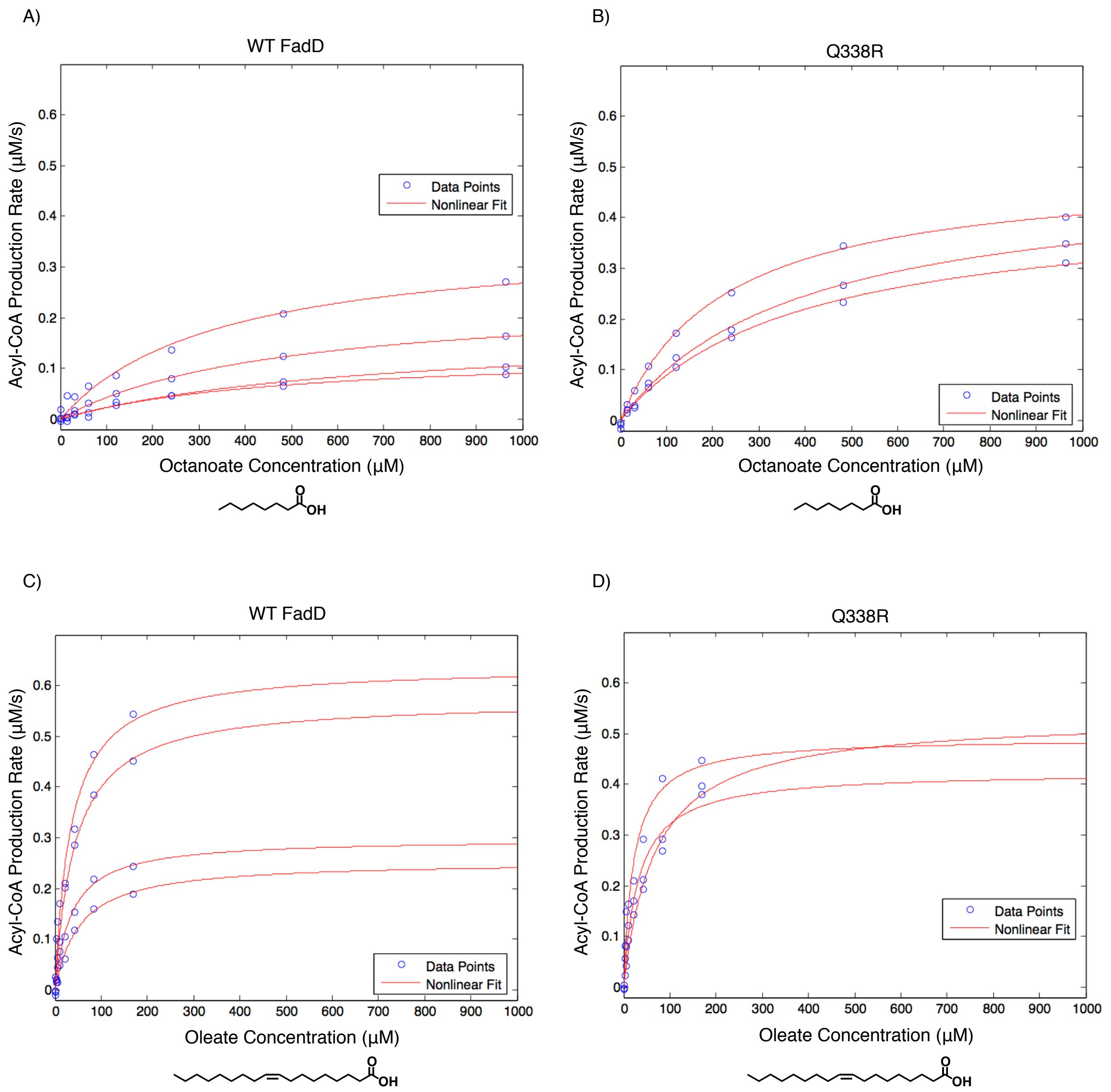

Figure S2: Sample rate vs. substrate concentration curves for the AMP production assay. Blue circles indicate rates determined from independent purifications and red lines are nonlinear fits to the Michaelis Menten equation for wild-type FadD and mutant Q338R using octanoate (A,B) and oleate (C,D) as substrates.

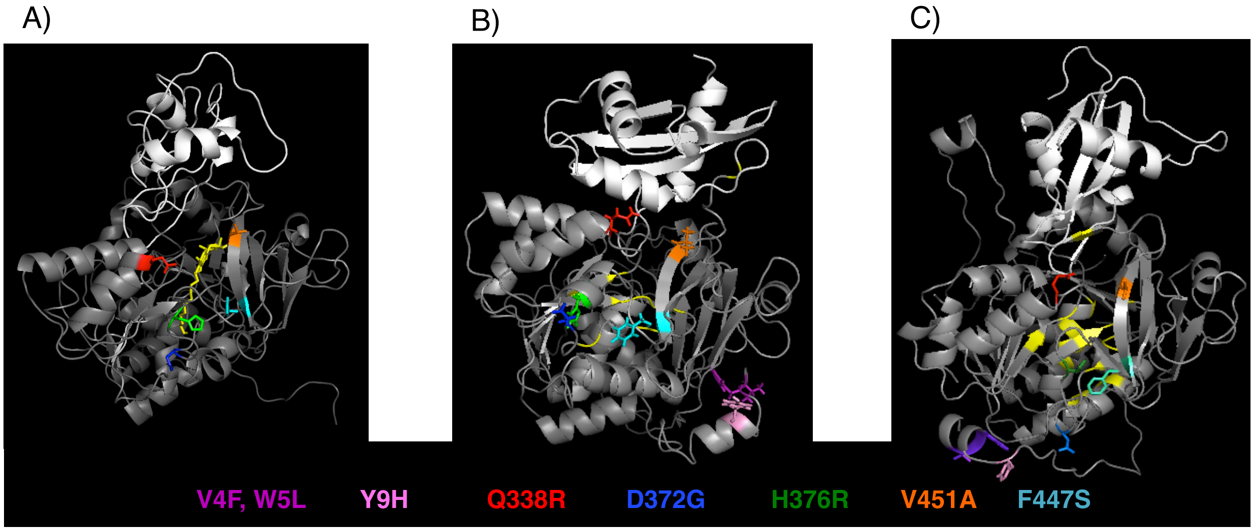

Figure S3. FadD Homology Modeling. Three separate FadD homology models were generated using (i) The SWISS-MODEL Homology modeling server (Arnold et al. 2006; Benkert et al. 2011; Biasini et al. 2014) and the *Thermus thermophilus* structure as the template, (ii) the I-TASSER server (Roy et al. 2010; Yang et al. 2015; Zhang 2008), and (iii) SAM-T08 (Karchin et al. 2004; Karchin et al. 2003; Karplus 2009; Karplus & Hu 2001; Karplus et al. 2001; Karplus et al. 2003; Karplus et al. 2005; Shackelford & Karplus 2007). Models were visualized in PyMOL with large N-terminal domain in gray, smaller C-terminal domain in white, and fatty acid binding pocket or myristoyl-AMP (overlayed from the *Thermus Thermophillus* structure) in yellow (Hisanaga et al. 2004; Shackelford & Karplus 2007). Residues whose mutation results in increased growth rate on octanoate are color-coded according to the identity of the mutation (text below models).

References

Arnold K, Bordoli L, Kopp J, and Schwede T. 2006. The SWISS-MODEL workspace: a web-based environment for protein structure homology modelling. *Bioinformatics* 22:195-201.

Benkert P, Biasini M, and Schwede T. 2011. Toward the estimation of the absolute quality of individual protein structure models. *Bioinformatics* 27:343-350.

Biasini M, Bienert S, Waterhouse A, Arnold K, Studer G, Schmidt T, Kiefer F, Cassarino TG, Bertoni M, Bordoli L, and Schwede T. 2014. SWISS-MODEL: modelling protein tertiary and quaternary structure using evolutionary information. *Nucleic Acids Research* 42:W252-W258.

Hisanaga Y, Ago H, Nakagawa N, Hamada K, Ida K, Yamamoto M, Hori T, Arii Y, Sugahara M, Kuramitsu S, Yokoyama S, and Miyano M. 2004. Structural basis of the substrate-specific two-step catalysis of long chain fatty acyl-CoA synthetase dimer. *The Journal of biological chemistry* 279:31717-31726.

Karchin R, Cline M, and Karplus K. 2004. Evaluation of local structure alphabets based on residue burial. *Proteins* 55:508-518.

Karchin R, Cline M, Mandel-Gutfreund Y, and Karplus K. 2003. Hidden Markov models that use predicted local structure for fold recognition: alphabets of backbone geometry. *Proteins* 51:504-514.

Karplus K. 2009. SAM-T08, HMM-based protein structure prediction. *Nucleic Acids Research* 37:W492-497.

Karplus K, and Hu B. 2001. Evaluation of protein multiple alignments by SAM-T99 using the BAliBASE multiple alignment test set. *Bioinformatics* 17:713-720.

Karplus K, Karchin R, Barrett C, Tu S, Cline M, Diekhans M, Grate L, Casper J, and Hughey R. 2001. What is the value added by human intervention in protein structure prediction? *Proteins* Suppl 5:86-91.

Karplus K, Karchin R, Draper J, Casper J, Mandel-Gutfreund Y, Diekhans M, and Hughey R. 2003. Combining local-structure, fold-recognition, and new fold methods for protein structure prediction. *Proteins* 53 Suppl 6:491-496.

Karplus K, Katzman S, Shackleford G, Koeva M, Draper J, Barnes B, Soriano M, and Hughey R. 2005. SAM-T04: what is new in protein-structure prediction for CASP6. *Proteins* 61 Suppl 7:135-142.

Roy A, Kucukural A, and Zhang Y. 2010. I-TASSER: a unified platform for automated protein structure and function prediction. *Nature Protocols* 5:725-738.

Shackelford G, and Karplus K. 2007. Contact prediction using mutual information and neural nets. *Proteins* 69 Suppl 8:159-164.

Yang JY, Yan RX, Roy A, Xu D, Poisson J, and Zhang Y. 2015. The I-TASSER Suite: protein structure and function prediction. *Nature Methods* 12:7-8.

Zhang Y. 2008. I-TASSER server for protein 3D structure prediction. *Bmc Bioinformatics* 9.
